# Association Between Peripheral IL-2^+^Th1/CD4^+^Tregs Axis Imbalance and Dysthyroid Optic Neuropathy in Thyroid Eye Disease

**DOI:** 10.3390/jcm15135283

**Published:** 2026-07-06

**Authors:** Zelu Wang, Zhenyu Piao, Tianyuan Li, Jia Zhang, Xiaoxia Li, Liang Fu, Mingwei Zhao, Wenzhen Yu, Lvzhen Huang, Fan Su

**Affiliations:** 1 Department of Ophthalmology, Peking University People’s Hospital, Beijing 100044, China; wangzelu.love@163.com (Z.W.);; 2 Key Laboratory of Ocular Disease and Optometry Science, Peking University People’s Hospital, Beijing 100044, China

**Keywords:** peripheral blood immune microenvironment, Thyroid Eye Disease (TED), dysthyroid optic neuropathy (DON), IL-2^+^Th1, CD4^+^Tregs

## Abstract

**Background/Objective:** Dysthyroid Optic Neuropathy (DON) is a severe complication of Thyroid Eye Disease (TED) leading to irreversible visual impairment. Its pathogenesis remains unclear, and early predictive tools are lacking. The study aims to investigate peripheral immune characteristics associated with DON, focusing on the IL-2^+^Th1/CD4^+^Tregs axis. **Methods:** A retrospective study was conducted in 37 TED patients, including DON (*n* = 22) and non-DON (*n* = 15) groups. Peripheral blood immune cell subsets were quantified using flow cytometry. Clinical data and peripheral blood immune indicators including T cell subsets, B cell subsets, T helper (Th) cell subsets, and regulatory T (Treg) cells populations were analyzed. Correlation and logistic regression analyses were applied to evaluate associations between immune indicators and DON. Receiver operating characteristic (ROC) analysis was used to assess the discriminatory performance of candidate variables and exploratory combined models. **Results:** Patients with DON showed higher IL-2^+^Th1 levels and lower CD4^+^Tregs levels compared with non-DON patients, along with an increased IL-2^+^Th1/CD4^+^Tregs ratio. Age and clinical activity score also differed significantly between groups. The IL-2^+^Th1/CD4^+^Tregs axis showed significant alterations associated with DON. The exploratory logistic regression model combining immune and clinical indicators showed potential discriminatory ability in differentiating DON from non-DON patients. **Conclusions:** This study identifies an imbalance between IL-2^+^Th1 and CD4^+^Tregs as a potential immune signature associated with DON. Integration of immune and clinical features may provide an exploratory framework for risk stratification in TED. Further prospective studies with larger cohorts are warranted to validate these findings.

## 1. Introduction

TED, as one of the most common extrathyroidal manifestations of Graves’ disease (GD), is an autoimmune disease centered on inflammatory processes [1,2]. Recent studies have further refined the immunopathogenesis of TED, highlighting the complex interplay between adaptive immune responses and orbital tissue remodeling [3]. In addition to the well-established role of autoantibody-mediated activation of orbital fibroblasts via the insulin-like growth factor-1 receptor (IGF-1R) and thyroid-stimulating hormone receptor (TSHR), accumulating evidence suggests that T cell–mediated immunity plays a central role in both disease initiation and progression. Elevated Th1-associated cytokines, including IL-2 and IFN-γ, together with impaired regulatory T cell function, may contribute to sustained orbital inflammation and tissue expansion. These findings suggest that systemic immune dysregulation is closely linked to disease severity in TED.

DON represents the most severe sight-threatening manifestation of TED [4,5,6]. The joint consensus issued by the American Thyroid Association (ATA) and the European Thyroid Association (ETA) in 2022 identified DON as one of the most vision-threatening and severe complications of TED [5]. The primary pathogenic mechanism involves mechanical compression of the optic nerve by edematous soft tissue at the orbital apex, rather than primary optic neuritis [5]. The 2021 consensus from the European Group on Graves’ Orbitopathy (EUGOGO) classified DON as the sole TED subtype necessitating urgent intervention [6]. Typical manifestations of DON include decreased visual acuity, dyschromatopsia, visual field defects, optic disc edema or pallor, and relative afferent pupillary defect (RAPD) [7,8]. Magnetic resonance imaging (MRI), computed tomography (CT) and other imaging studies typically demonstrate extraocular muscle enlargement compressing the optic nerve, abnormal optic nerve course, or orbital apex crowding due to retro-orbital tissue accumulation [9]. The tensile force caused by chronic exophthalmos may also contribute to secondary optic nerve injury [8]. Although mechanical compression at the orbital apex has traditionally been considered the primary mechanism, increasing evidence indicates that inflammatory and immunological factors may also contribute to its development [10]. Patients with DON often exhibit more active and dysregulated immune profiles compared with non-DON cases, suggesting that immune imbalance may contribute to progression to optic nerve involvement. However, the immunological characteristics underlying DON remain incompletely understood. In early-stage DON, most patients show significant symptom improvement with proper treatment, but delayed intervention risks permanent blindness. Current diagnosis primarily depends on clinical manifestations supported by imaging, yet suffers from subjective bias and poor early imaging sensitivity. In recent years, significant advances have been made in the management of moderate-to-severe TED. The introduction of targeted biologic therapies, particularly teprotumumab targeting IGF-1R, has reshaped the therapeutic landscape and demonstrated efficacy in reducing proptosis and disease activity. Other immunomodulatory agents, including rituximab and tocilizumab, have been investigated for their potential to modulate inflammatory pathways in refractory cases [11]. Orbital decompression remains a cornerstone surgical intervention [5,12,13,14,15], particularly in DON, where rapid reduction of orbital pressure is required to prevent irreversible optic nerve damage. An effective method of identifying patients at risk of DON or predicting disease severity are still lacking.

Although immune dysregulation has been implicated in TED pathogenesis, the specific peripheral immune signature associated with DON and its relationship with clinical disease severity have not been fully elucidated. Whether an imbalance between related immune cells and inflammatory cytokines contributes to DON development in a clinically meaningful manner remains unclear.

The present study aimed to investigate peripheral immune characteristics in TED patients with and without DON, with a particular focus on the IL-2^+^Th1/CD4^+^Tregs axis. We further sought to evaluate the association between immune profiles and DON status, and to explore the potential value of integrating immune and clinical parameters for disease stratification using exploratory statistical and logistic regression models.

## 2. Methods

### 2.1. Patient Recruitment

Thirty-seven patients with TED were enrolled from Peking University People’s Hospital and classified into two groups according to the presence of DON: 22 in the DON Group and 15 in the non-DON Group. Written informed consent was obtained, and peripheral blood samples were collected. All procedures complied with the Declaration of Helsinki and were approved by the Ethics Committee of Peking University People’s Hospital (Approval No.: 2024PHB344-001).

For clarity, patients were classified into the DON group and non-DON group according to the presence or absence of clinically diagnosed dysthyroid optic neuropathy. All patients in the DON group fulfilled the diagnostic criteria for DON. Paired pre- and post-treatment peripheral blood samples were available in a subset of 9 TED patients who received clinical treatment and had complete follow-up data. Treatment selection in the follow-up cohort was based on clinical severity and visual function status rather than thyroid functional status in accordance with current EUGOGO and ATA-ETA guideline-based management recommendations [5,6]. Orbital decompression was arranged promptly after initial consultation for DON patients with clinically significant optic nerve involvement and risk of visual deterioration, whereas non-DON patients without visual impairment received teprotumumab infusion therapy. Pre-treatment samples were collected before clinical intervention, and post-treatment samples were collected at least one month after treatment during follow-up.

### 2.2. Diagnosis and Exclusion Criteria

The diagnosis of patients with TED was in line with the Bartley & Gorman criteria published in 1995 [16] and the 2021 consensus from the EUGOGO [6]: presence of eyelid retraction, combined with one of thyroid dysfunction, exophthalmos, optic nerve dysfunction, or extraocular muscle involvement, for patients without eyelid retraction, concurrent presence of thyroid dysfunction plus one of exophthalmos, extraocular muscle involvement, or optic nerve dysfunction was required, exclusion of other orbital diseases.

The diagnosis of patients with DON required meeting at least 2 of the following criteria [7,8,9]: best-corrected visual acuity (BCVA) <0.8, color vision disturbance, visual field defect, pallor or edema of the optic disc, positive RAPD, abnormal results of pattern visual evoked potential (VEP) examination, imaging examinations such as orbital MRI or CT showing extraocular muscle enlargement compressing the optic nerve, abnormal optic nerve course, or orbital apex crowding.

Patients were excluded if they were pregnant or lactating females, had prior orbital surgery, experienced severe infections within the past year, had received systemic glucocorticoid pulse therapy, immunosuppressive agents, biological therapy, orbital radiotherapy, or previous orbital surgery before baseline peripheral blood sampling or had comorbidities significantly affecting immune function, including rheumatic immune diseases, malignancies, or hematologic disorders. Patients with isolated radiological orbital apex crowding or optic nerve compression without clinical manifestations meeting the diagnostic criteria for DON were excluded from this study.

### 2.3. Basic Information Collection

Patient data included sex, age, smoking history, serum thyrotropin receptor autoantibodies (TRAb) level, thyroid function status, degree of orbital protrusion, orbital pressure, and extraocular muscle thickness. Ocular examination findings were recorded to calculate the CAS. Ocular examination findings were recorded to calculate the CAS, which assesses the activity of TED based on seven indicators: spontaneous retro-orbital pain, pain with eye movement, eyelid hyperemia and edema, conjunctival hyperemia and edema, and inflammatory signs such as caruncle swelling and redness. One point was awarded for each positive sign, and patients with a total score ≥3/7 were diagnosed as having active TED [6].

### 2.4. Detection Items and Assay Methods

A total of 6 laboratory tests were performed after collecting peripheral blood from the subjects, including complete blood count, thyroid function determination, Th cell subset detection, Treg cells subset detection, B cell subset detection and T cell subset detection (detailed items are listed in Supplementary Table S1). All laboratory tests were performed in the Department of Clinical Laboratory of Peking University People’s Hospital using standardized and automated clinical diagnostic platforms as part of routine hospital practice. Complete blood count parameters were measured using a Beckman Coulter automated hematology analyzer (Beckman Coulter, Inc., Brea, CA, USA). Thyroid function tests and serum TRAb levels were determined using a Roche cobas e 601 electrochemiluminescence immunoassay system (Roche Diagnostics GmbH, Mannheim, Germany). Peripheral blood lymphocyte subsets were quantified using an Agilent NovoCyte flow cytometer (Agilent Technologies, Inc., Santa Clara, CA, USA) with standardized clinical flow cytometry protocols. All assays were performed according to institutional standard operating procedures and the manufacturers’ instructions. No custom-designed or research-only immunophenotyping panels were used in this study.

Peripheral blood samples were collected in EDTA anticoagulant tubes. For Th cell subset analysis, cells were stimulated with ionomycin in the presence of brefeldin A, followed by red blood cell lysis, fixation, permeabilization, and intracellular cytokine staining using routine clinical laboratory reagents. Th cell subsets were identified using intracellular cytokines (TNF-α, IFN-γ, IL-2, IL-4, IL-17). Treg cells were defined as CD4^+^CD25^+^FoxP3^+^ T cells. B cell subsets were identified using standard clinical immunophenotyping panels. Detailed commercial information for these routine clinical reagents was not available because these assays were performed as standardized clinical tests by the hospital clinical laboratory. Data acquisition was performed using a standardized clinical flow cytometry system, and analysis was conducted using FlowJo software (version 10.10; FlowJo LLC, Ashland, OR, USA). Results were expressed as percentages of parent populations according to routine clinical reporting standards.

### 2.5. Statistical Analysis

For consistent table formatting, data were presented as mean ± SD. Continuous variables were first assessed for normality using the Shapiro–Wilk test. Data with an approximately normal distribution in both groups were compared using Welch’s *t*-test. Variables that did not meet the normality assumption in either group were presented as median and interquartile range and compared using the Mann–Whitney U test. Categorical variables were presented as counts and percentages and compared using the chi-square test or Fisher’s exact test, as appropriate.

Spearman correlation analysis was used to evaluate the association between all immune characteristics including IL-2^+^Th1 and CD4^+^Tregs. Spearman-based partial correlation analysis was further performed after adjustment for age and CAS. Fisher’s r-to-z transformation was used as an exploratory method to compare correlation coefficients between the DON and non-DON subgroups.

Logistic regression was used to evaluate factors associated with DON. Univariate logistic regression was first performed, followed by multivariable logistic regression models adjusting for potential clinical confounders, including age, sex, CAS, smoking history, and TRAb level. For exploratory model evaluation, predicted probabilities were generated from the logistic regression model and ROC curve analysis was then used to assess apparent discriminatory performance. The area under the curve (AUC), 95% confidence interval (95% CI), optimal cut-off, sensitivity, and specificity were reported. ROC analysis was used to evaluate model performance rather than to construct the model itself.

For the paired follow-up analysis, pre- and post-treatment peripheral blood samples were available from nine TED patients. As a retrospective study, the exact interval between treatment and follow-up blood sampling was not completely uniform among patients. Paired pre- and post-treatment comparisons were performed after assessing the normality of paired differences using the Shapiro–Wilk test.

All statistical tests were two-sided, and *p* < 0.05 was considered statistically significant.

## 3. Results

### 3.1. Preliminary Analysis of Baseline Clinical and Peripheral Immune Characteristics

To comprehensively characterize baseline clinical and peripheral immune profiles in TED patients, we first performed an integrated analysis of all measured variables between DON and non-DON groups.

**Figure 1:**
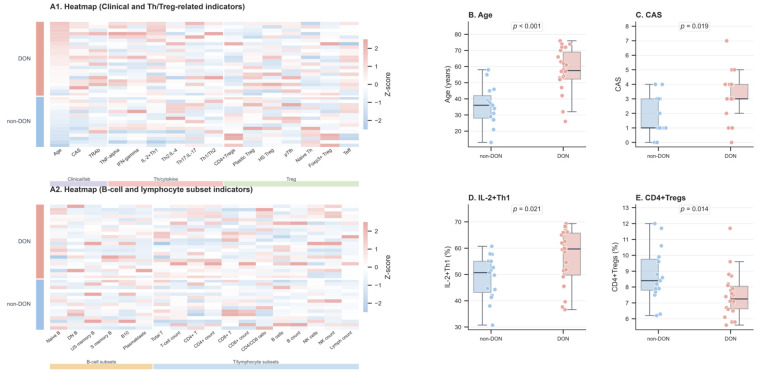
**Baseline clinical and peripheral blood immune characteristics of TED patients with and without DON.** (**A1**,**A2**) Heatmaps showing the distribution patterns of peripheral blood clinical and immune indicators in TED patients with DON and non-DON. A1 displays clinical parameters and Th/Treg-related immune indicators, and A2 displays B-cell subsets and lymphocyte subset indicators. All variables were standardized using Z-scores prior to visualization. Patients were stratified by group (DON vs. non-DON), indicated by the left-side annotation bar. (**B**) Comparison of age between DON and non-DON groups. (**C**) Comparison of CAS between the two groups. (**D**) Comparison of IL-2^+^Th1 levels between the two groups. (**E**) Comparison of CD4^+^Tregs levels between the two groups. (The colored horizontal bars below the heatmaps indicate different categories of measured variables, including clinical/laboratory parameters, Th/cytokine-related indicators, Treg-related indicators, B-cell subsets, and T/lymphocyte subset indicators. DON and non-DON groups are shown in red and blue, respectively. Continuous variables were compared using Welch’s *t*-test or Mann–Whitney U test according to data distribution. Exact *p* values are shown in each panel).

As shown in Figure 1(A1,A2), heatmap analysis of standardized peripheral blood and immune parameters revealed distinct distribution patterns between the two groups. Patients with DON exhibited a broader dysregulation across multiple immune cell subsets and cytokine-related indicators compared with non-DON patients, suggesting a systemic immune imbalance associated with DON status.

In the clinical comparison, DON patients were significantly older than non-DON patients (*p* < 0.001, Welch’s *t*-test; Table 1; Figure 1B). The CAS was significantly higher in the DON group than in the non-DON group (*p* = 0.019, Mann–Whitney U test; Table 1; Figure 1C), indicating increased disease activity in patients with optic nerve involvement. Smoking history did not differ significantly between the two groups. TRAb levels were numerically higher in the DON group than in the non-DON group, although the difference did not reach statistical significance (*p* = 0.089, Mann–Whitney U test; Table 1). Regarding key immune subsets, IL-2^+^Th1 levels were significantly elevated in DON patients compared with non-DON patients (*p* = 0.021, Mann–Whitney U test; Table 1; Figure 1D), whereas CD4^+^Tregs were significantly decreased in the DON group (*p* = 0.014, Mann-Whitney U test; Table 1; Figure 1E), and a higher IL-2^+^Th1/CD4^+^Tregs ratio (Welch’s *t*-test) than the non-DON group. These findings suggest a shift toward a pro-inflammatory immune profile accompanied by relative impairment of Treg cells activity in DON. Collectively, these results demonstrate that DON is associated with distinct alterations in both clinical severity and peripheral immune signatures, characterized by enhanced Th1-associated immune activation and reduced Treg cells proportions. These findings provide the phenotypic basis for subsequent multivariable regression and predictive modeling analyses.

### 3.2. Integrated Correlation Analysis of Immune Parameters in TED

To systematically investigate the relationship between immune cell subsets in TED, we first performed a global exploratory correlation analysis of peripheral immune parameters. The overall correlation structure among immune indicators is presented in Supplementary Figure S1, which reveals a broad immune interaction network and supports the presence of coordinated alterations in multiple lymphocyte subsets.

**Figure 2:**
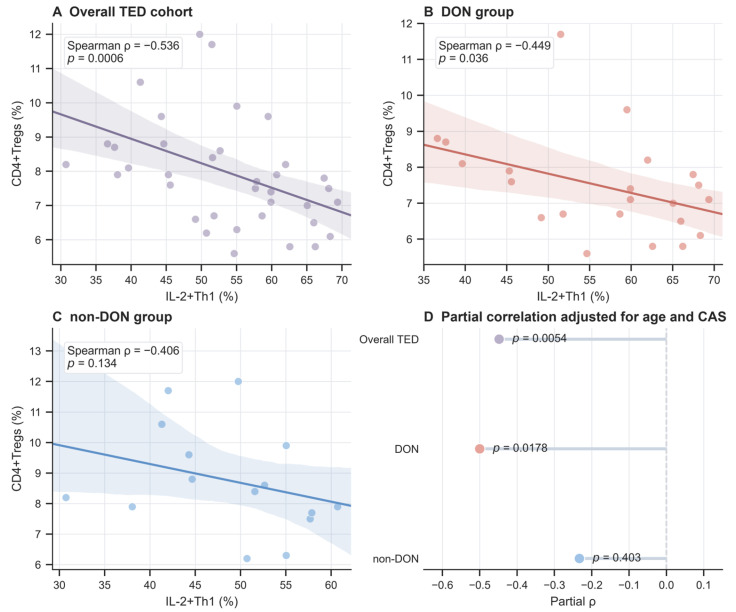
**Correlation between IL-2^+^Th1 and CD4^+^Tregs in TED patients.** (**A**) Spearman correlation analysis between IL-2^+^Th1 and CD4^+^Tregs in the overall TED cohort. (**B**) Spearman correlation analysis in the DON group. (**C**) Spearman correlation analysis in the non-DON group. (**D**) Partial correlation analysis between IL-2^+^Th1 and CD4^+^Tregs after adjustment for age and CAS. (The colors in panels A–C correspond to the group colors shown in panel D: purple indicates the overall TED cohort, red indicates the DON group, and blue indicates the non-DON group. Each point represents an individual subject. Solid lines indicate linear regression fits with 95% CI. Correlation coefficients (ρ) and corresponding *p* values are shown within each panel. Partial correlation coefficients were calculated using Spearman-based methods adjusting for age and CAS).

Based on these global patterns, we next focused on the key immunological axis between IL-2^+^Th1 cells and CD4^+^Tregs, which showed a consistent inverse relationship. As shown in Figure 2, IL-2^+^Th1 levels were negatively correlated with CD4^+^Tregs in the overall cohort as well as in subgroup analyses, with varying degrees of association strength. To further evaluate whether this relationship was independent of potential confounding factors, we performed Spearman-based partial correlation analysis adjusting for age and CAS. The results are summarized in Table 2. After adjustment, the negative association between IL-2^+^Th1 and CD4^+^Tregs remained significant in the overall cohort and in the DON group, whereas this association was attenuated and no longer statistically significant in the non-DON group.

Although the correlation coefficient was numerically stronger in the DON group, formal comparison using Fisher’s r-to-z transformation showed no statistically significant difference between the DON and non-DON groups (z = −0.143, *p* = 0.886). Therefore, this should be interpreted with caution.

### 3.3. Logistic Regression Analysis of Factors Associated with DON

To evaluate clinical and immune factors associated with DON, we first performed univariate logistic regression analysis. The candidate variables included age, male sex, CAS, smoking history, TRAb, IL-2^+^Th1, CD4^+^Tregs, and the IL-2^+^Th1/CD4^+^Tregs ratio. In the univariate analysis, age, CAS, IL-2^+^Th1, CD4^+^Tregs, and the IL-2^+^Th1/CD4^+^Tregs ratio showed associations with DON, whereas male sex, smoking history, and TRAb were not statistically significant.

**Figure 3:**
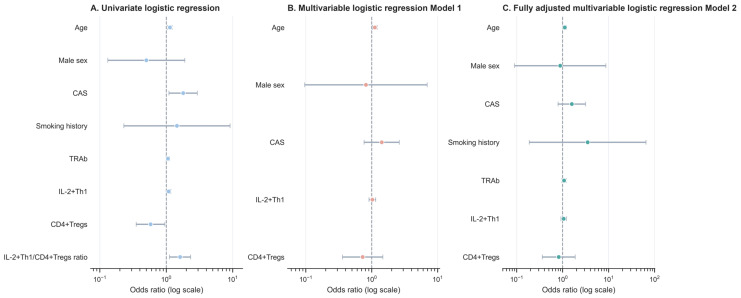
**Logistic regression analysis of factors associated with DON.** (**A**) Univariate logistic regression analysis of candidate clinical and immune variables, including age, male sex, CAS, smoking history, TRAb, IL-2^+^Th1, CD4^+^Tregs, and the IL-2^+^Th1/CD4^+^Tregs ratio. (**B**) Multivariable logistic regression Model 1 adjusted for age, male sex, CAS, IL-2^+^Th1, and CD4^+^Tregs. (**C**) Fully adjusted multivariable logistic regression Model 2 including age, male sex, CAS, smoking history, TRAb, IL-2^+^Th1, and CD4^+^Tregs. (Different colors indicate different regression models: blue for univariate logistic regression, pink for multivariable logistic regression Model 1, and green for fully adjusted multivariable logistic regression Model 2. Dots indicate odds ratios, and horizontal lines represent 95% CI. The vertical dashed line indicates an odds ratio of 1. Odds ratios are displayed on a logarithmic scale).

Considering the baseline differences in age and CAS between the DON and non-DON groups, we further performed multivariable logistic regression analyses. Model 1 included age, male sex, CAS, IL-2^+^Th1, and CD4^+^Tregs. Model 2 was further adjusted for smoking history and TRAb, and therefore included age, male sex, CAS, smoking history, TRAb, IL-2^+^Th1, and CD4^+^Tregs. After multivariable adjustment, age remained significantly associated with DON, whereas IL-2^+^Th1 and CD4^+^Tregs showed the expected direction of association but did not reach statistical significance. These results suggest that IL-2^+^Th1 and CD4^+^Tregs should be interpreted as exploratory immune features associated with DON rather than confirmed independent predictors. The logistic regression results are summarized in Table 3 and Figure 3.

In multivariable models, the statistical significance of IL-2^+^Th1 and CD4^+^Tregs was attenuated, suggesting potential confounding effects of age and disease activity.

### 3.4. Exploratory ROC Analysis

**Figure 4:**
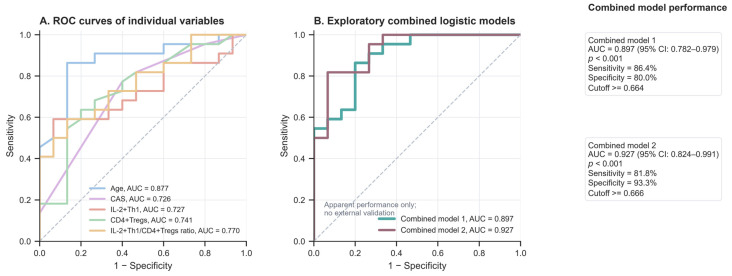
**Exploratory ROC analysis for distinguishing DON from non-DON TED patients.** (**A**) ROC curves of individual clinical and immune variables, including age, CAS, IL-2^+^Th1, CD4^+^Tregs, and the IL-2^+^Th1/CD4^+^Tregs ratio. (**B**) ROC curves of exploratory combined logistic regression models. Combined model 1 included age, CAS, IL-2^+^Th1, and CD4^+^Tregs. Combined model 2 included age, male sex, CAS, smoking history, TRAb, IL-2^+^Th1, and CD4^+^Tregs. (The diagonal dashed line represents the reference line for no discrimination. The AUC is shown for each individual variable or combined model. Sensitivity and specificity of the combined models were calculated at the optimal cut-off determined by the Youden index. The ROC results reflect apparent discriminatory performance in the present cohort and were not externally validated).

To further assess the apparent discriminatory performance of candidate clinical and immune variables, we performed exploratory ROC curve analysis for distinguishing DON from non-DON TED patients. Among individual variables, age showed the highest AUC (AUC = 0.877), followed by the IL-2^+^Th1/CD4^+^Tregs ratio (AUC = 0.770), CD4^+^Tregs (AUC = 0.741), IL-2^+^Th1 (AUC = 0.727), and CAS (AUC = 0.726). (Figure 4A).

We then evaluated two exploratory combined logistic regression models. Combined model 1 included age, CAS, IL-2^+^Th1, and CD4^+^Tregs. This model showed an AUC of 0.897 (95% CI: 0.782–0.979, *p* < 0.001; Figure 4B), with a sensitivity of 86.4% and a specificity of 80.0% at the optimal cut-off of 0.664. Combined model 2 included age, male sex, CAS, smoking history, TRAb, IL-2^+^Th1, and CD4^+^Tregs. This fully adjusted exploratory model showed an AUC of 0.927 (95% CI: 0.824–0.991, *p* < 0.001; Figure 4B), with a sensitivity of 81.8% and a specificity of 93.3% at the optimal cut-off of 0.666.

Although the combined models demonstrated higher apparent discriminatory performance than individual variables, these results should be interpreted cautiously because of the limited sample size and lack of external validation. Therefore, these models should be regarded as exploratory logistic regression-based models rather than clinically validated prediction tools. The ROC curves are shown in Figure 4.

### 3.5. Paired Pre- and Post-Treatment Changes in Immune Indicators

To further explore dynamic changes in immune indicators after treatment, we analyzed paired pre- and post-treatment peripheral blood samples from nine TED patients with available follow-up data.

**Figure 5:**
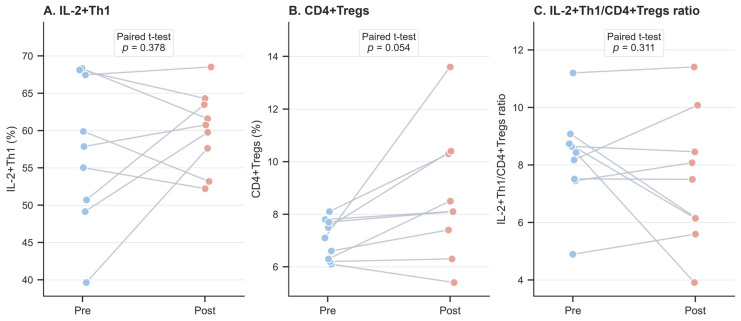
**Paired pre- and post-treatment changes in immune indicators in TED patients with follow-up data (*n* = 9).** (**A**) IL-2^+^Th1 levels before and after treatment. (**B**) CD4^+^Tregs levels before and after treatment. (**C**) IL-2^+^Th1/CD4^+^Tregs ratio before and after treatment. (Each line represents paired measurements from an individual patient. Pre-treatment values are shown in blue and post-treatment values in red, with gray lines indicating paired connections. Statistical comparisons were performed using paired *t*-test. Exact *p* values are indicated in each panel).

As shown in Figure 5, IL-2^+^Th1 levels showed no statistically significant change after treatment (*p* = 0.378, paired *t*-test; Figure 5A). CD4^+^Tregs showed a trend toward increase after treatment (*p* = 0.054, paired *t*-test; Figure 5B). The IL-2^+^Th1/CD4^+^Tregs ratio did not show a statistically significant change (*p* = 0.311, paired *t*-test; Figure 5C).

Given the limited number of paired cases and the non-uniform follow-up sampling intervals, these findings should be interpreted as exploratory observations rather than conclusive evidence of treatment-related immune changes.

## 4. Discussion

As a pivotal human immunomodulatory cytokine, IL-2 has been identified as an essential signal for the survival and functional maintenance of Tregs [17,18,19,20]. IL-2 exerts dual biological effects: low-dose IL-2 selectively engages the high-affinity trimeric IL-2 receptor complex (CD25^+^CD122^+^CD132^+^), and evidence from multiple clinical trials in autoimmune diseases demonstrates its capacity to selectively expand Treg populations [17,21,22], thereby safely and effectively attenuating disease activity. Conversely, high-dose IL-2 preferentially activates effector T cells, a mechanism closely associated with antitumor immunotherapy [18]. Our results demonstrated a significantly elevated proportion of IL-2^+^Th1 cells in patients with DON, which may modulate CD4^+^Tregs function through paracrine IL-2, thus impacting systemic immune regulation. Such sustained overproduction of IL-2 not only strengthened the differentiation and proliferation of effector T cells but also disrupted the fine balance between proinflammatory and anti-inflammatory immune subsets. Treg cells, particularly CD4^+^Tregs subsets, play a central role in maintaining autoimmune homeostasis by persistently suppressing autoreactive CD4^+^/CD8^+^ T cells, B cells, and dendritic cells, thus preventing the onset of autoimmune pathology [23,24,25]. Systematic review studies have further demonstrated that the proportion of Treg cells in both orbital tissue and peripheral blood of patients with TED is reduced [25], accompanied by impaired suppressive function, which exhibits a negative correlation with disease activity [26,27].

Among the 37 patients enrolled in this study, statistically significant differences were observed in age and CAS between the DON Group and the non-DON Group. These findings reaffirm that age and CAS are risk factors for the development of DON [5,6,15,28,29]. We observed that Th cells producing proinflammatory cytokines, particularly IL-2^+^Th1, were significantly increased in the peripheral blood of TED patients, while CD4^+^Treg cells were decreased, and the proportions of IL-2^+^Th1 and CD4^+^Treg differed markedly between the DON and non-DON Group (Table 1; Figure 1). These findings indicate that peripheral immune profiles may mirror intraorbital inflammation in TED and may serve as indicators of disease severity and activity.

A significant negative correlation between IL-2^+^Th1 and CD4^+^Tregs was observed in the overall TED cohort. This inverse association remained significant in the DON subgroup, whereas it was not statistically significant in the non-DON subgroup (Table 2; Figure 2). After adjusting for age and CAS, the association remained robust in the overall cohort and DON group but not in non-DON patients, suggesting that the relationship between IL-2^+^Th1 and CD4^+^Tregs may be more stable in DON patients, although formal subgroup comparison did not demonstrate a statistically significant difference. This may be related to the limited sample size, subgroup variability, and baseline clinical heterogeneity. Future studies with larger cohorts and more refined stratification, including patients with radiological orbital apex crowding but without clinically evident DON, may help determine whether this immune relationship changes during DON progression.

Logistic regression analysis (Table 3; Figure 3) demonstrated that age, CAS, and IL-2^+^Th1 were associated with DON in univariate models, whereas CD4+Tregs showed a protective association. However, after adjustment in multivariable models, the associations of immune-related variables were attenuated, suggesting that their effects may be partially confounded by clinical disease activity and demographic factors such as age. These findings indicate that peripheral immune markers may reflect overall disease severity yet cannot serve as independent predictors of DON.

To further explore the discriminatory potential of these variables, an exploratory ROC analysis was performed. The combined model incorporating immune and clinical parameters showed improved discrimination compared with individual variables, with an AUC of 0.897 (Figure 4). This suggests that the integrated model may reflect discriminatory patterns in the present dataset. Given the retrospective design, limited sample size, and lack of external validation, these findings should be interpreted cautiously and considered hypothesis-generating. Further validation in independent, larger cohorts is required to assess the robustness and generalizability of this observation.

The observed increase in CD4^+^Tregs in our paired follow-up analysis may reflect overall clinical improvement and reduced inflammatory burden, as restoration of Treg homeostasis has been associated with disease remission and immune recovery in autoimmune conditions [30]. In thyroid-associated ophthalmopathy, alterations in Treg frequency and function have been reported and may partially recover following treatment [31]. Immunosuppressive therapies have also been shown to restore immune balance, including regulatory T-cell compartments, in autoimmune diseases. Importantly, orbital decompression primarily alleviates mechanical orbital apex crowding rather than directly modulating systemic immune pathways [6]. Therefore, postoperative changes in Tregs are more likely attributable to reduced disease activity, concomitant medical therapy, postoperative immune re-equilibration, or systemic inflammatory resolution, rather than a direct immunological effect of surgery itself.

Several immune-related mechanisms may be involved in the IL-2^+^Th1/CD4^+^Tregs imbalance observed in TED. IL-2 is a key cytokine involved in T cell homeostasis and may influence the balance between effector T cell activation and regulatory T cell maintenance [32]. In this context, a relative increase in Th1-associated activity together with reduced Treg proportion may reflect a shift toward a pro-inflammatory immune state. Aging-related thymic involution and impaired T cell output may contribute to reduced regulatory capacity in peripheral immunity [33]. Emerging evidence also suggests that Treg stability may be sensitive to inflammatory and metabolic stress, including pathways related to ferroptosis [34,35]. These processes may collectively influence the balance between immune activation and suppression in TED. Given the cross-sectional design of this study, these associations should be interpreted as correlational rather than causal.

Based on our current results, the IL-2^+^Th1/CD4^+^Tregs axis imbalance should be interpreted as an exploratory immune indicator. Future prospective studies with larger cohorts, standardized treatment protocols, predefined response criteria, and serial immune monitoring are needed to determine whether baseline, post-treatment, or dynamic changes in the IL-2^+^Th1/CD4^+^Tregs ratio can predict clinical responsiveness in TED or DON. Differences in treatment modalities between the two follow-up patient groups may also have introduced a certain degree of heterogeneity, which could potentially influence the comparability of the results. However, given the limited sample size of the follow-up cohort, subgroup analyses stratified by treatment type were not performed in the present study. This issue should be addressed in future studies with larger sample sizes and more homogeneous treatment designs.

This is the first study to identify a distinct peripheral immune signature characterized by an imbalance between IL-2^+^Th1 and CD4^+^Tregs in TED patients with DON. By integrating clinical activity parameters with immune profiling, we demonstrate that this Th1/Treg-associated axis is associated with disease severity and may contribute to the discrimination between DON and non-DON TED patients. Researchers have attempted to explore its pathogenesis by analyzing immune profile changes in periorbital tissues [7], and some progress has been made. Nevertheless, obtaining intra-orbital tissues is difficult, which hinders large-scale screening of patients with TED for the prevention and treatment of DON. The present study investigated the peripheral blood immune microenvironment in DON patients, which enables the use of more easily acquired specimens. By analyzing changes in multiple immune indicators, this study achieved certain progress in identifying approaches that may guide clinical diagnosis and treatment and help predict disease progression, thereby contributing to more precise and standardized management of optic neuropathy. An exploratory multivariable model integrating immune and clinical parameters also demonstrated a certain discriminatory performance. Although the relevant conclusions remain preliminary, the findings that peripheral immune dysregulation may play a key role in the pathological process of optic neuropathy also provide a potential immunological framework for future disease risk stratification and mechanistic studies.

Several limitations should be acknowledged. The study was a single-center retrospective study with a relatively small sample size, which may limit the generalizability of the findings and increase the risk of selection bias. Current study failed to incorporate certain risk factors associated with TED, including selenium status. Although we adjusted for key clinical covariates such as age and CAS in the correlation and regression analyses, residual confounding could not be fully excluded. In addition, biochemical hyperthyroidism was present in a subset of DON patients, while thyroid function status was not regarded as a major confounding factor driving the observed intergroup immune differences, which may be affected by the small sample size needing to be validated in subsequent studies. The follow-up analysis was limited by the small number of paired samples and heterogeneous treatment strategies; therefore, the post-treatment changes in immune indicators should be interpreted as exploratory rather than conclusive evidence of treatment response prediction.

In future study designs, prospective multicenter trials should be considered, with larger sample sizes and standardized treatment protocols. Studies should incorporate predefined outcome measures, including visual acuity, visual field, CAS, imaging parameters, and treatment outcomes, while also accounting for additional potential confounders such as selenium levels, in order to more accurately evaluate whether baseline levels or dynamic changes in the IL-2^+^Th1/CD4^+^Tregs axis can predict clinical treatment response. Follow-up records of patients after treatment should also be stratified according to thyroid functional status and different treatment modalities. Serial immune monitoring before and after treatment, combined with cytokine profiling and, where feasible, validation using orbital tissue samples or experimental models, may further help clarify whether this immune pathway is directly involved in the pathogenesis of DON.

Overall, these findings suggest an association between peripheral immune alterations and DON in TED. Further studies are required to determine their biological and clinical relevance.

## 5. Conclusions

This is the first study to identify an imbalance between IL-2^+^Th1 and CD4^+^Tregs in TED patients with DON, suggesting an association between peripheral immune dysregulation and optic nerve involvement. This achievement is expected to help shift the current diagnostic approach, which relies solely on imaging findings and empirical treatment, into an active prevention and management model based on precise prediction using immune markers. The exploratory model constructed by integrating immune indicators and clinical parameters demonstrated potential value in differential diagnosis; however, due to its single-center design and limited sample size, the findings remain preliminary and have not yet been clinically validated. Future larger-scale prospective studies are warranted, with more comprehensive pre- and post-treatment data comparisons, to further confirm the role of the IL-2^+^Th1/CD4^+^Tregs axis in thyroid-associated ophthalmopathy-related optic neuropathy.

## Figures and Tables

**Table 1:** **Baseline clinical and immune characteristics of TED patients with and without DON.** (Data are presented as mean ± standard deviation or *n* (%). Group comparisons were performed using Welch’s *t*-test or Mann–Whitney U test for continuous variables according to data distribution, and chi-square or Fisher’s exact test for categorical variables.).

Clinical Information	Total TED (*n* = 37)	DON Group (*n* = 22)	Non-DON Group (*n* = 15)	*p*-Value
**No. of subjects**	37	22	15	—
**Age, years**	48.89 ± 16.75	57.86 ± 13.28	35.73 ± 12.05	<0.001
**Male sex, *n* (%)**	16 (43.2%)	8 (36.4%)	8 (53.3%)	0.306
**Smoking history, *n* (%)**	6 (16.2%)	4 (18.2%)	2 (13.3%)	1.000
**CAS**	2.68 ± 1.67	3.23 ± 1.57	1.87 ± 1.51	0.019
**TRAb, IU/L (0–1.75)**	12.67 ± 13.52	16.07 ± 14.59	7.69 ± 10.29	0.089
**IL-2^+^Th1, % (35.9–53.1)**	53.43 ± 10.32	56.58 ± 10.48	48.80 ± 8.41	0.021
**CD4^+^Tregs, % (5–10)**	7.99 ± 1.65	7.47 ± 1.40	8.75 ± 1.73	0.014
**IL-2^+^Th1/CD4^+^Tregs ratio**	7.06 ± 2.29	7.90 ± 2.27	5.83 ± 1.75	0.004
**Thyroid function status**				0.487
**Biochemical hyperthyroid, *n* (%)**	7 (18.9%)	4 (18.2%)	3 (20.0%)	
**Biochemical hypothyroid, *n* (%)**	2 (5.4%)	1 (4.5%)	1 (6.7%)	
**Euthyroid range, *n* (%)**	14 (37.8%)	7 (31.8%)	7 (46.7%)	
**Subclinical hyperthyroid pattern, *n* (%)**	8 (21.6%)	7 (31.8%)	1 (6.7%)	
**Subclinical hypothyroid pattern, *n* (%)**	6 (16.2%)	3 (13.6%)	3 (20.0%)	

**Table 2:** **Correlation and partial correlation between IL-2^+^Th1 and CD4^+^Tregs in TED patients.** (Data are presented as Spearman correlation coefficients (ρ) and partial correlation coefficients. Partial correlation analysis was performed after adjustment for age and CAS using Spearman rank-based methods. The difference between subgroup Spearman correlation coefficients in the DON and non-DON groups was assessed using Fisher’s r-to-z transformation and was not statistically significant (z = −0.143, *p* = 0.886)).

Group	Spearman ρ	*p* Value	Partial ρ (Adjusted for Age and CAS)	*p* Value
**Total TED (*n* = 37)**	−0.536	0.0006	−0.448	0.0054
**DON group (*n* = 22)**	−0.449	0.036	−0.500	0.0178
**non-DON group (*n* = 15)**	−0.406	0.134	−0.233	0.403

**Table 3:** **Univariate and multivariable logistic regression analysis of factors associated with DON.** (Data are presented as OR with 95% CI. Univariate logistic regression was performed for initial screening of candidate variables. Multivariable Model 1 was adjusted for age, male sex, CAS, IL-2^+^Th1, and CD4^+^Tregs. Multivariable Model 2 additionally included smoking history and TRAb. The IL-2^+^Th1/CD4^+^Tregs ratio was evaluated only in univariate analysis to avoid collinearity with its component variables. A two-sided *p* < 0.05 was considered statistically significant. NS indicates not statistically significant.).

Variable	Univariate OR (95% CI)	*p*-Value	Multivariable Model 1 OR (95% CI)	*p*-Value	Multivariable Model 2 OR (95% CI)	*p*-Value
**Age**	1.133 (1.062–1.240)	<0.001	1.11 (1.03–1.21)	0.010	1.11 (1.03–1.21)	0.010
**CAS**	1.796 (1.140–3.090)	0.012	1.58 (0.94–2.87)	0.083	1.61 (0.96–2.95)	0.071
**Male sex**	NS	—	NS	—	NS	—
**Smoking history**	NS	—	—	—	NS	—
**TRAb**	NS	—	—	—	NS	—
**IL-2^+^Th1**	1.086 (1.013–1.180)	0.006	1.05 (0.92–1.20)	0.460	1.05 (0.92–1.20)	0.460
**CD4^+^Tregs**	0.579 (0.326–0.908)	0.020	0.82 (0.36–1.87)	0.642	0.82 (0.36–1.87)	0.642

## Data Availability

The data presented in this study are available on request from the corresponding author due to patient privacy and ethical data protection restrictions.

## Supplementary Materials

The following supporting information can be downloaded at: https://www.mdpi.com/article/10.3390/jcm15135283/s1, Figure S1: Correlation heatmap of peripheral immune parameters in TED patients. (This heatmap illustrates the pairwise correlations among peripheral blood immune parameters in the overall TED cohort. Correlation coefficients were calculated using Spearman rank correlation analysis. Positive correlations are shown in shades of red, while negative correlations are shown in shades of blue. The intensity of the color reflects the strength of the correlation. Statistically significant correlations are indicated where applicable.); Table S1. Peripheral blood laboratory parameters and immune cell subsets. (All parameters were measured using standardized clinical laboratory platforms in the Department of Clinical Laboratory of Peking University People’s Hospital.).

## Abbreviations

The following abbreviations are used in this manuscript:

95% CI	95% confidence interval
ATA	American Thyroid Association
AUC	Area Under the Curve
BCVA	Best-corrected visual acuity
CAS	Clinical Activity Score
CD4^+^Tregs	CD4^+^ regulatory T cells
CT	Computed tomography
DON	Dysthyroid Optic Neuropathy
ETA	European Thyroid Association
EUGOGO	European Group on Graves’ Orbitopathy
FDR	False Discovery Rate
GD	Graves’ disease
GO	Graves’ ophthalmopathy
IGF-1R	Insulin-like growth factor-1 receptor
IL-2^+^Th1	Interleukin-2–positive T helper 1 cells
MRI	Magnetic resonance imaging
OR	Odds ratio
RAPD	Relative afferent pupillary defect
ROC	Receiver Operating Characteristic
TED	Thyroid Eye Disease
TRAb	Thyrotropin receptor autoantibodies
TSHR	Thyroid-stimulating hormone receptor

## References

[1] Wang L., Chen L. (2025). Emerging therapeutic approaches in graves’ ophthalmopathy: An update on pharmacological interventions. Front. Immunol..

[2] Zhang P., Zhu H. (2022). Cytokines in Thyroid-Associated Ophthalmopathy. J. Immunol. Res..

[3] Cieplińska K., Niedziela E., Kowalska A. (2024). Immunological Processes in the Orbit and Indications for Current and Potential Drug Targets. J. Clin. Med..

[4] Dolman P.J. (2020). Dysthyroid optic neuropathy: Evaluation and management. J. Endocrinol. Investig..

[5] Burch H.B., Perros P., Bednarczuk T., Cooper D.S., Dolman P.J., Leung A.M., Mombaerts I., Salvi M., Stan M.N. (2022). Management of Thyroid Eye Disease: A Consensus Statement by the American Thyroid Association and the European Thyroid Association. Thyroid.

[6] Bartalena L., Kahaly G.J., Baldeschi L., Dayan C.M., Eckstein A., Marcocci C., Marinò M., Vaidya B., Wiersinga W.M. (2021). The 2021 European Group on Graves’ orbitopathy (EUGOGO) clinical practice guidelines for the medical management of Graves’ orbitopathy. Eur. J. Endocrinol..

[7] Ma Q., Hai Y., Duan Y., Yu G., Song C., Huang S., Huang A., Zhu Y., Shen Y., Huang Z. (2025). Inflammatory profiling and immune cell infiltration in dysthyroid optic neuropathy: Insights from bulk RNA sequencing. Front. Immunol..

[8] Miśkiewicz P., Rutkowska B., Jabłońska A., Krzeski A., Trautsolt-Jeziorska K., Kęcik D., Milczarek-Banach J., Pirko-Kotela K., Samsel A., Bednarczuk T. (2016). Complete recovery of visual acuity as the main goal of treatment in patients with dysthyroid optic neuropathy. Endokrynol. Pol..

[9] Tu Y., Xu M., Kim A.D., Wang M.T.M., Pan Z., Wu W. (2021). Modified endoscopic transnasal orbital apex decompression in dysthyroid optic neuropathy. Eye Vis..

[10] Zhang M., Liang L., Hu H., Tang C., Huang Q., Long J. (2025). Correlation between orbital immune cell subsets and clinical activity in thyroid eye disease. Ann. Endocrinol..

[11] Scarabosio A., Surico P.L., Singh R.B., Tereshenko V., Musa M., D’Esposito F., Russo A., Longo A., Gagliano C., Agosti E. (2024). Thyroid Eye Disease: Advancements in Orbital and Ocular Pathology Management. J. Pers. Med..

[12] Baczewska N., Alexopoulou O., Constantinescu S.M., Daumerie C., Coutel M., Boschi A., Burlacu M.C. (2025). Factors Associated with Response to Intravenous Glucocorticoids in Active Moderate-to-Severe Thyroid Eye Disease. Thyroid.

[13] Pelewicz-Sowa M., Miśkiewicz P. (2023). Dysthyroid optic neuropathy: Emerging treatment strategies. J. Endocrinol. Investig..

[14] Wang M., Jiang X., Geng J., Hui S., Li D. (2023). Outcomes of Patients with Dysthyroid Optic Neuropathy Treated with Intravenous Corticosteroids and/or Orbital Decompression Surgery: A Systematic Review and Meta-analysis. J. Clin. Endocrinol. Metab..

[15] Wiersinga W.M., Eckstein A.K., Žarković M. (2025). Thyroid eye disease (Graves’ orbitopathy): Clinical presentation, epidemiology, pathogenesis, and management. Lancet. Diabetes Endocrinol..

[16] Bartley G.B., Gorman C.A. (1995). Diagnostic Criteria for Graves’ Ophthalmopathy. AM. J. Ophthalmol..

[17] Ye C., Brand D., Zheng S.G. (2018). Targeting IL-2: An unexpected effect in treating immunological diseases. Signal Transduct. Target. Ther..

[18] Overwijk W.W., Tagliaferri M.A., Zalevsky J. (2021). Engineering IL-2 to Give New Life to T Cell Immunotherapy. Annu. Rev. Med..

[19] Xu L., Song X., Su L., Zheng Y., Li R., Sun J. (2019). New therapeutic strategies based on IL-2 to modulate Treg cells for autoimmune diseases. Int. Immunopharmacol..

[20] DeOca K.B., Moorman C.D., Garcia B.L., Mannie M.D. (2020). Low-Zone IL-2 Signaling: Fusion Proteins Containing Linked CD25 and IL-2 Domains Sustain Tolerogenic Vaccination in vivo and Promote Dominance of FOXP3(+) Tregs in vitro. Front. Immunol..

[21] Whangbo J.S., Kim H.T., Nikiforow S., Koreth J., Alho A.C., Falahee B., Kim S., Dusenbury K., Fields M.J., Reynolds C.G. (2019). Functional analysis of clinical response to low-dose IL-2 in patients with refractory chronic graft-versus-host disease. Blood Adv..

[22] Lorenzon R., Ribet C., Pitoiset F., Aractingi S., Banneville B., Beaugerie L., Berenbaum F., Cacoub P., Champey J., Chazouilleres O. (2024). The universal effects of low-dose interleukin-2 across 13 autoimmune diseases in a basket clinical trial. J. Autoimmun..

[23] Abeles I., Palma C., Meednu N., Payne A.S., Looney R.J., Anolik J.H. (2024). B Cell-Directed Therapy in Autoimmunity. Annu. Rev. Immunol..

[24] Mishra S., Liao W., Liu Y., Yang M., Ma C., Wu H., Zhao M., Zhang X., Qiu Y., Lu Q. (2021). TGF-β and Eomes control the homeostasis of CD8+ regulatory T cells. J. Exp. Med..

[25] Huang Y., Fang S., Li D., Zhou H., Li B., Fan X. (2019). The involvement of T cell pathogenesis in thyroid-associated ophthalmopathy. Eye.

[26] Liu Z., Ke S.-R., Shi Z.-X., Zhou M., Sun L., Sun Q.-H., Xiao B., Wang D.-L., Huang Y.-J., Lin J.-S. (2024). Dynamic transition of Tregs to cytotoxic phenotype amid systemic inflammation in Graves’ ophthalmopathy. JCI Insight.

[27] Rodríguez-Muñoz A., Vitales-Noyola M., Ramos-Levi A., Serrano-Somavilla A., González-Amaro R., Marazuela M. (2016). Levels of regulatory T cells CD69(+)NKG2D(+)IL-10(+) are increased in patients with autoimmune thyroid disorders. Endocrine.

[28] Wang X., Ye H., Chen R., Yang S., Zhang T., Xiao W., Yang H. (2023). HbA1c: An independent risk factor for dysthyroid optic neuropathy. Front. Endocrinol..

[29] Vermot-Desroches V., Thia-Soui-Tchong K., Raymond P., Filip A., Orgiazzi J., Jouanneau E., Froment Tilikete C., Borson-Chazot F., Manet R., Abeillon Du Payrat J. (2023). Prognostic factors for significant 6-month recovery in dysthyroid optic neuropathy in a tertiary center: A series of 69 eyes in 38 patients. Ann. Endocrinol..

[30] Lu L., Barbi J., Pan F. (2017). The regulation of immune tolerance by FOXP3. Nat. Rev. Immunol..

[31] Yuan X., Li H., Wang F. (2026). The therapeutic revolution in thyroid eye disease: From orbital radiotherapy to teprotumumab and AI. Front. Med..

[32] Cheng H., Nan F., Ji N., Ma X., Zhang J., Liang H., Zhang W., Nakatsukasa H., Zhang H., Jin W. (2025). Regulatory T cell therapy promotes TGF-β and IL-6-dependent pro-inflammatory Th17 cell generation by reducing IL-2. Nat. Commun..

[33] Elyahu Y., Monsonego A. (2021). Thymus involution sets the clock of the aging T-cell landscape: Implications for declined immunity and tissue repair. Ageing Res. Rev..

[34] Ma C., Li H., Lu S., Li X. (2024). Thyroid-associated ophthalmopathy and ferroptosis: A review of pathological mechanisms and therapeutic strategies. Front. Immunol..

[35] Xu C., Sun S., Johnson T., Qi R., Zhang S., Zhang J., Yang K. (2021). The glutathione peroxidase Gpx4 prevents lipid peroxidation and ferroptosis to sustain Treg cell activation and suppression of antitumor immunity. Cell Rep..

