# Supplementary material for: Association Between Peripheral IL-2+Th1/CD4+Tregs Axis Imbalance and Dysthyroid Optic Neuropathy in Thyroid Eye Disease
Source: J Clin Med. 2026 Jul 6;15(13):5283. doi: 10.3390/jcm15135283 (PMC13363665; doi:10.3390/jcm15135283)
Supplement: Supplementary file 1 [file jcm-15-05283-s001.zip › jcm-4356534-supplementary.pdf]

Category	Parameters
**Complete blood count (CBC)**	White blood cell count (WBC); Lymphocyte absolute count; Red blood cell count (RBC); Hemoglobin (Hb); Platelet count (PLT)
**Thyroid function and autoantibodies**	Free triiodothyronine (FT3); Free thyroxine (FT4); Triiodothyronine (T3); Thyroxine (T4); Thyroid-stimulating hormone (TSH); Thyroglobulin antibody (TgAb); Thyroid peroxidase antibody (TPOAb); TSH receptor antibody (TRAb); Anti-TSH receptor antibody (TSHR-Ab)
**Th cell subsets**	TNF-α^+ Th1 cells; IFN-γ^+ Th1 cells; IL-2^+ Th1 cells; IL-4^+ Th2 cells; IL-17^+ Th17 cells; Follicular helper T cells (pTfh); Naïve T helper cells; Effector T helper cells (Teff)
**Treg cell subsets**	$CD4^+$ Treg cells; $FoxP3^+$ Treg cells; Plastic Treg cells; Highly suppressive Treg cells
**B cell subsets**	Naïve B cells; Double-negative B cells; Unswitched memory B cells; Switched memory B cells; B10 cells; Plasmablasts
**T cell subsets (include NK cell subsets)**	Total T cells ($CD3^+$); Total lymphocyte T cell count; $CD4^+$ T helper/inducer cells; $CD4^+$ T cell absolute count; $CD8^+$ cytotoxic/suppressor T cells; $CD8^+$ T cell absolute count; CD4/CD8 ratio; NK cell percentage; NK cell absolute count; Total lymphocyte absolute count

Supplementary Table S1. Peripheral blood laboratory parameters and immune cell subsets. (All parameters were measured using standardized clinical laboratory platforms in the Department of Clinical Laboratory of Peking University People's Hospital.)

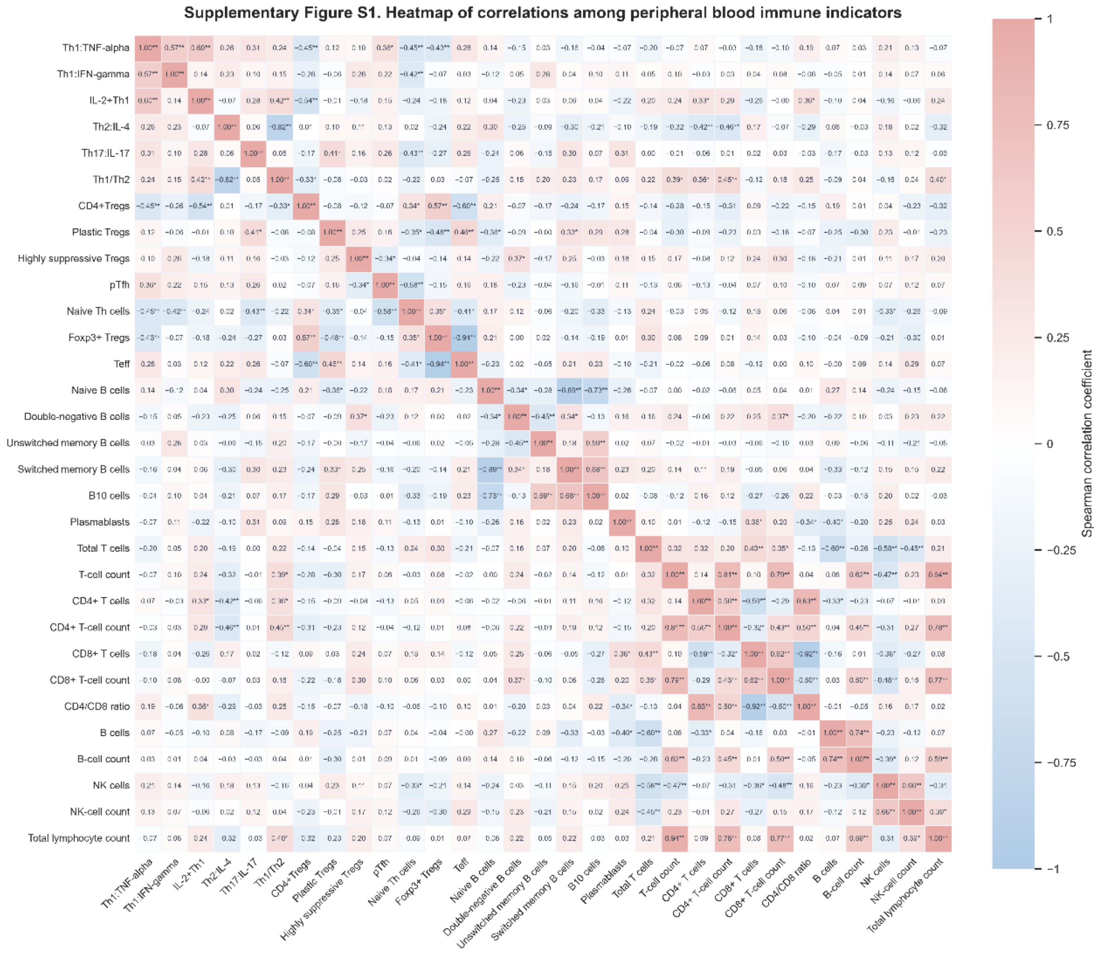

Supplementary Figure S1. Correlation heatmap of peripheral immune parameters in TED patients. (This heatmap illustrates the pairwise correlations among peripheral blood immune parameters in the overall TED cohort. Correlation coefficients were calculated using Spearman rank correlation analysis. Positive correlations are shown in shades of red, while negative correlations are shown in shades of blue. The intensity of the color reflects the strength of the correlation. Statistically significant correlations are indicated where applicable.)
